# Elevated WNT signaling and compromised Hedgehog signaling due to *Evc2* loss of function contribute to the abnormal molar patterning

**DOI:** 10.3389/fdmed.2022.876015

**Published:** 2022-08-01

**Authors:** Honghao Zhang, Afriti Chinoy, Paymon Mousavi, Aubrey Beeler, Ke’ale Louie, Crystal Collier, Yuji Mishina

**Affiliations:** Department of Biologic and Materials Sciences & Prosthodontics, School of Dentistry, University of Michigan, Ann Arbor, MI, United States

**Keywords:** EVC2, limbin, molar, WNT, cilia, supernumerary tooth

## Abstract

Ellis-van Creveld (EVC) syndrome is an autosomal recessive chondrodysplasia. The affected individuals bear a series of skeleton defects, congenital heart septum anomalies, midfacial defects, and dental defects. Previous studies using *Evc* or *Evc2* mutant mice have characterized the pathological mechanism leading to various types of congenital defects. Some patients with EVC have supernumerary tooth; however, it is not known yet if there are supernumerary tooth formed in *Evc* or *Evc2* mutant mice, and if yes, what is the pathological mechanism associated. In the present study, we used *Evc2* mutant mice and analyze the pattern of molars in *Evc2* mutant mice at various stages. Our studies demonstrate that *Evc2* loss of function within the dental mesenchymal cells leads to abnormal molar patterning, and that the most anterior molar in the *Evc2* mutant mandible represents a supernumerary tooth. Finally, we provide evidence supporting the idea that both compromised Hedgehog signaling and elevated WNT signaling due to *Evc2* loss of function contributes to the supernumerary tooth formation.

## Introduction

First reported in 1940, Ellis-van Creveld (EVC) syndrome is a rare congenital disease with an estimated occurrence of 1 in 20,000–60,000 births (1, 2). Genetic studies have linked two thirds of EVC syndrome occurrences to genetic mutations in either EVC or EVC2 (aka LIMBIN) (2–5). Typical clinical signs of EVC include dwarfism, postaxial polydactyly, congenital heart defects, and dysplasia of ectoderm-derived tissues such as hair and nails (2, 6). Additionally, genetic mutations or single nucleotide polymorphisms of EVC2 have been identified in Japanese brown cattle, Tyrolean Grey cattle, and penguins, thereby suggesting an evolutionarily conserved function of EVC2/LIMBIN (7–9).

In addition to the aforementioned clinical signs, EVC syndrome also presents with various dental abnormalities including natal teeth, delayed eruption, hypodontia, supernumerary teeth, conical-shaped teeth, taurodontism, and hypoplastic enamel (10, 11). These alterations in tooth eruption, number, and morphology in patients with EVC syndrome suggest that EVC2 plays an important role across multiple stages of tooth development.

Tooth development is a complicated process involving coordinated interactions between epithelium and mesenchymal-derived tissues (12). Organogenesis initiates with thickening of the dental epithelium and progresses through the bud stage, cap stage, and bell stage (12). Next, a single layer of epithelial and mesenchymal cells proliferate and differentiate into ameloblasts and odontoblasts, respectively, to generate dental mineralized tissues in the crown (13). Following the crown formation, the apical epithelia tissues elongate and give to a bilayer structure, Hertwig’s epithelial root sheath (HERS). Then continuously growing of HERS guides the growth of dental mesenchymal tissue, which leads to formation of root and support of further growing of tooth root (14). Across these stages, multiple signaling pathways including Hedgehog, BMP, WNT, and FGF coordinate to ensure that each tooth achieves the correct morphology (15). In mammals, molars align in a single row. Mouse mandibular molar row development initiates from transiently existing premolars, MS and R2 (16). At E14.5, along with regress of MS and R2, the first molar (M1) formed (16). *In vivo* and *ex vivo* reveals a model that anterior molar influences the initiation and development of the next one both in size and shape (16–18). However, full characterization of detailed molecular signaling network is still needed to provide knowledge fundamental to understand the hypodontia and supernumerary tooth formation.

EVC syndrome is categorized as a ciliopathy due to the ciliary localization of proteins encoded by the causative genes of EVC syndrome, EVC and EVC2/LIMBIN (3). Molecular studies have demonstrated that EVC and EVC2 form a protein complex, which locates to the bottom of primary cilium and is required for full transduction of Hedgehog signaling (3, 19–21). We recently generated *Evc2* mutant mouse lines (21) and delineated the pathological mechanisms leading to the both dwarfism and midfacial hypoplasia (21–27). We also demonstrated that compromised Hedgehog signaling due to *Evc2* loss of function within dental mesenchymal tissue secondarily delays ameloblast differentiation and is responsible for hypoplastic enamel formation in *Evc2* mutant mice (26).

Similar to human patients with EVC syndrome (28), *Evc* mutant mice present with abnormal tooth morphology (29). However, detailed descriptions of molar dysmorphology and supernumerary molar identity in *Evc* mutant mice has not been determined (29). Here we used *Evc2* loss-of-function mutant mice to characterize molar pattern and shape. Our results indicated that *Evc2* loss of function leads to abnormal patterning of molars. Several lines of evidence suggest that the most anterior molars are supernumerary teeth. The abnormal molar patterning is due to *Evc2* loss of function within dental mesenchyme. In addition to decreased Hedgehog signaling, we found concurrently elevated WNT signaling in molars from *Evc2* mutant mice. Our data supports the hypothesis that decreased Hedgehog signaling and elevated WNT signaling contributes to the abnormal molar patterning observed in *Evc2* mutant mice.

## Materials and methods

### Animal models

Mice in the present studies were maintained and used in compliance with the Institutional Animal Care and Use Committee (IACUC) of the University of Michigan in accordance with the National Institutes of Health Guidelines for Care and Use of Animals in research, and all experimental procedures were approved by the IACUC of the University of Michigan (PRO00009613). Generation of *Evc2* mutant mice (*Evc2* ^*ex12/+*^) and *Evc2* floxed mice (*Evc2* ^*fl/+*^) were as reported previously (21). The *Evc2* floxed mice were bred with *Sox2-*Cre mice (30) to generate mice carrying a Cre-recombined allele (*dE*). Deletion of *Evc2* in a neural crest-specific manner was achieved through crossing *Evc2* ^*fl/fl*^ mice with either *P0-Cre* mice (31) or *Wnt1-Cre* mice (32). WNT signaling reporter mice were from Jaxson 004623 (33). All mice were maintained in a mixed background of C57BL6/J and 129S6 and were crossed and maintained in our semi-closed mouse colony for at least 7 years. There are no samples exclusions in our studies.

### Micro-CT (μCT)

For micro-CT analysis, mouse heads at P28 were fixed in 4% paraformaldehyde in phosphate buffered saline followed by scanning at the University of Michigan using a micro-CT system (μCT100 Scanco Medical, Bassersdorf, Switzerland). Scan settings were as following: voxel size 12 μm, 55 kVp, 109 μA, 0.5 mm AL filter, and integration time 500 ms.

### Image acquisition, segmentation and surface models

To visualize the molars at postnatal day 8 (P8), the surface model for molars were generated based on the micro-CT data, using ITK-SNAP (open-source software developed by grants and contracts from the U.S. National Institutes of Health, www.itksnap.org), according to standard of segmentation using ITK-SNAP and previously described protocols (23, 34).

### Tooth germs dissection, mouse embryonic fibroblast (MEF) isolation and *in vitro* induction of WNT signaling

Mouse tooth germs were dissected from E18.5 mandibles. Under a dissection microscope, tooth germs were separated from surrounding tissues with forceps. For analysis of Hedgehog signaling and WNT signaling, total RNA was isolated from tooth germs homogenized in Trizol (Life Technology) according to standard protocols. Quantitative real-time PCR (Q-RT-PCR) was performed using Applied Biosystems ViiA7, with the following taqman probes: *Axin2*: Mm00443610_m1, *Lef1*: Mm00550265_m1, *Gli1*: Mm00494654_m1, *Ptch1*: Mm00436026_m1, and *Gapdh*: Mm99999915_g1. MEFs were prepared form E12.5 embryos and cultured in (DMEM with 10% Fetal bovine serum (FBS) according to previously described method (35). For arresting cell cycles and allowing growth of primary cilia, MEFs were cultured in MEF culture media (DMEM with 0.5% FBS) for at least 18 h. WNT signaling was induced by treating cells with WNT3A in concentration of 100 μg/ml for 18 h. Then total RNA was isolated from non-treated or treated cells. Then, 1 μg of total RNA was reverse transcribed using SuperScript Reverse Transcriptase (Life Technologies, Grand Island, NY, USA). Q-RT-PCR for examining induction of WNT signaling was performed in using Applied Biosystems ViiA7, with the following taqman probes: *Axin2*: Mm00443610_m1, *Lef1*: Mm00550265_m1, and *Gapdh*: Mm99999915_g1.

### Histology, and immunohistochemistry

Mouse mandibles were dissected out at P8, E18.5, E16.5, E15.5 or E14.5 and fixed in 4% paraformaldehyde (PFA). Subsequently, they were embedded in paraffin, sectioned parasagittally, and stained with hematoxylin and eosin for histologic observations according to standard histology procedure. For immunohistochemistry, dissected mandibles were fixed with 4% PFA and cryo-protected by 30% sucrose in PBS before being embedded parasagittally for cryosection. Sections were incubated overnight at 4 °C with antibody against SHH (Hybridoma Bank, University of Iowa), LEF1 (Cell signaling 2230). Sections were then incubated with secondary antibody (IgG-Alexa fluor 488 (A-32731), IgG-Alexa fluor 594 (A-21203)) at room temperature for 1 h. And subsequently mounted by Prolong Gold antifade mount with DAPI (P36935, Life Technology) and imaged under a confocal microscope (Nikon C1). The intensity of signaling was quantified using Image J. For intensity quantification, there are at least 40 cells were quantified and the average of all quantified cells were used to represent the biological sample. There are 4 pairs of controls and mutants were used for comparisons.

### Statistical analysis

Paired t test was conducted in all studies, which was performed in SPSS 27.0 (IBM Corp., Armonk, NY, USA). Error bars in the graph indicate standard deviation.

## Results

### *Evc2* mutant mice have abnormal molar patterning

**FIGURE 1:**
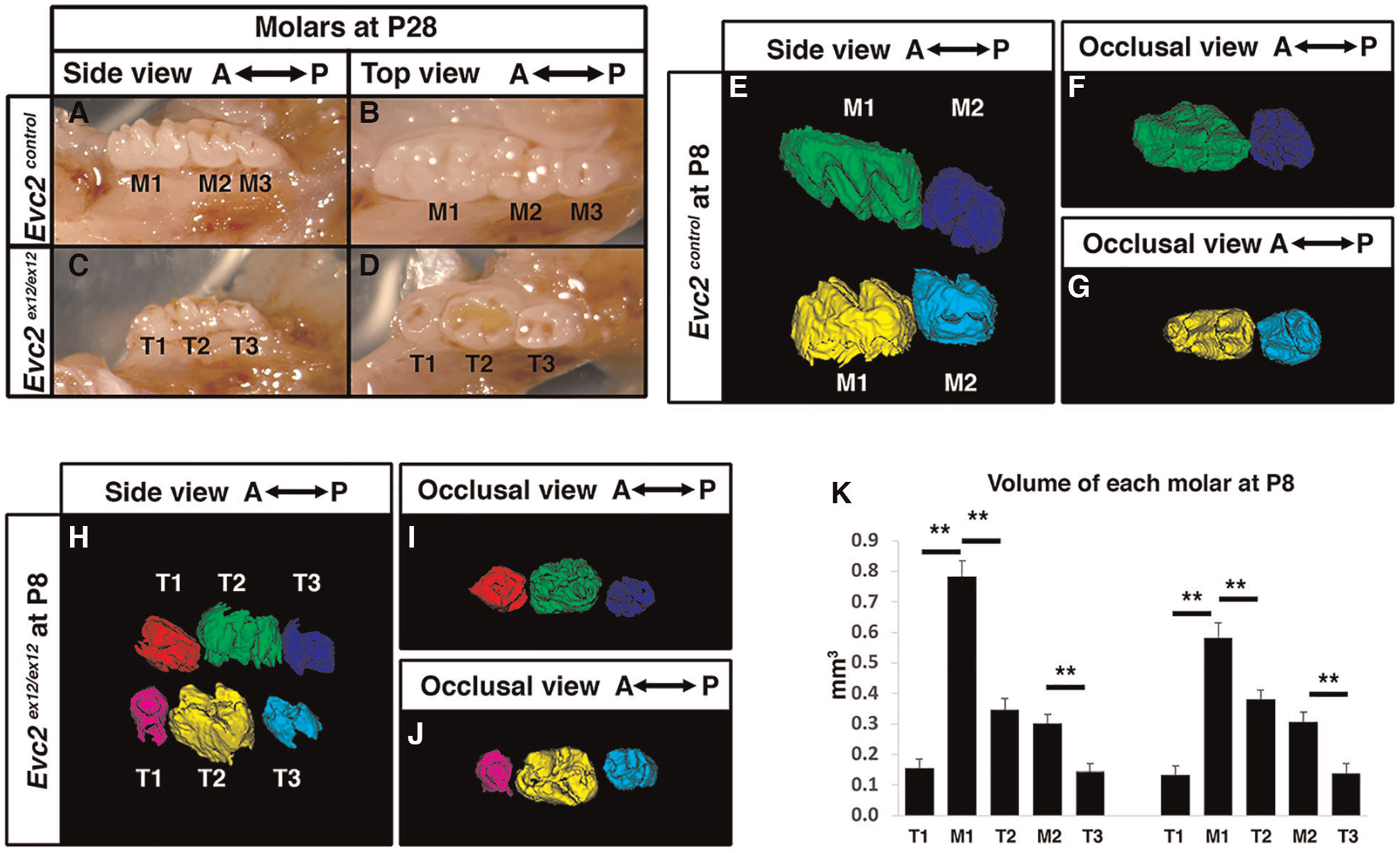
*Evc2* mutant mice have abnormal molar morphology. Lateral and occlusal images showing the gross morphology of postnatal day 28 (P28) mandibular molars from control (**A**,**B**) and *Evc2* mutant (**C**,**D**) mice. Molars from controls are marked as M1, M2, and M3 from anterior to posterior. Molars from *Evc2* mutants are marked as T1, T2, and T3 from anterior to posterior. CT-reconstructed molars at postnatal day 8 (P8) from control (**E**–**G**) and *Evc2* mutant (**H**–**J**) mice. Comparison of control vs. *Evc2* mutant molar volumes at P8 (**K**). *n* = 4, for maxilla, M1 vs T1, *p* < 0.01; M1 vs T2, *p* < 0.01; M2 vs T3, *p* < 0.01; for mandible, M1 vs T1, *p* < 0.01; M1 vs T2, *p* < 0.01; M2 vs T3, *p* < 0.01.

In contrast to alterations in tooth number reported in patients with EvC syndrome, we did not observe different numbers of molars in *Evc2* mutant mice (Figures 1A–D) (26). This occurred despite the presence of previously reported dental phenotypes including hypoplastic enamel and incisor shortening (26). However, molar patterning differed between *Evc2* mutants and their control counterparts at postnatal day 28 (P28). In control mice at P28, mandibular molars had a gradated pattern of decreasing crown size from anterior-to-posterior with a patterning of M1 as the biggest, then M2, and M3 as the smallest (Figures 1A,B). This is in contrast with the mandibular molars of *Evc2* mutant mice at P28 which showed no regular size gradations from anterior-to-posterior, with T2 as the biggest, then T3, and T1 as the smallest (Figures 1C,D). These observations were consistent with previous reports on both *Evc* and *Evc2* mutant mice (26, 29).

To understand the mechanism leading to abnormal patterning of molars in *Evc2* mutant mice, we then evaluate the molars at postnatal day 8 (P8), a stage when M1 and M2 are almost ready to erupt, but M3 is still with minimal levels of mineralization in control mice. As expected, in control mice at postnatal day 8 (P8), the M1 and M2 were mineralized, present, and nearing eruption while M3 had minimal levels of mineralization and were not present in scans (Figures 1E–G). In contrast, we observed three mineralized molar bodies, T1, T2, and T3, in the maxillae and mandibles of *Evc2* mutants (Figures 1H–J). Volumetric quantification of P8 molars in each arch corroborated P28 molar pattern abnormalities and indicated that M1 was larger than either T1 or T2 and that M2 was larger than T3 (Figure 1K). The observation of 3 molars in *Evc2* mutants at P8, however, led us to question the actual identity of each molar and the role of *Evc2* on molar pattern and morphology. We henceforth used neural-crest specific *Evc2* mutant mice to investigate these questions as these mice presented with identical molar phenotypes (see next section) to *Evc2* global mutants while circumventing problems associated with postnatal lethality and delayed growth (21).

### *Evc2* function within dental mesenchyme is critical for molar patterning

**FIGURE 2:**
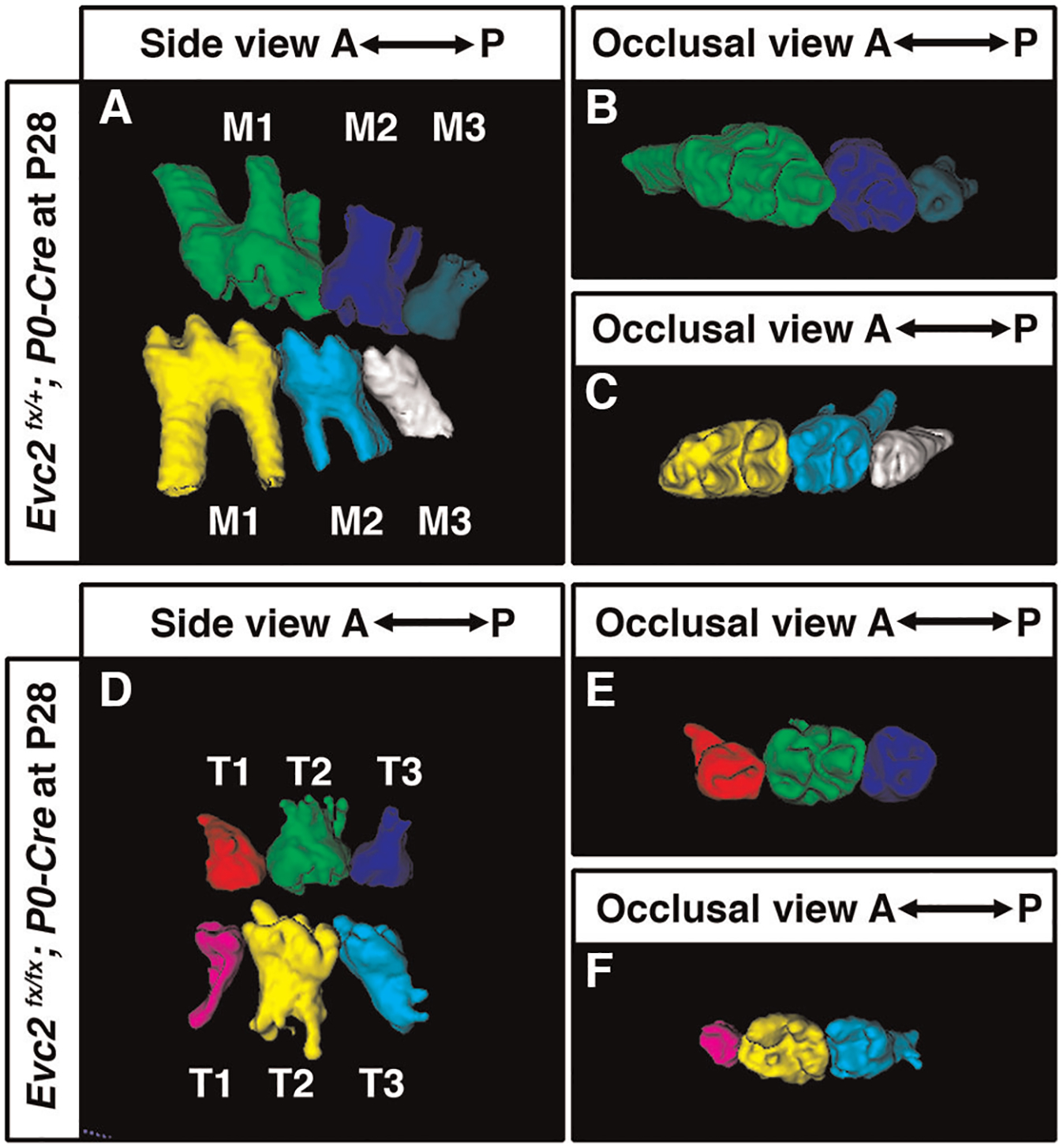
Molars at P28 from controls (**A**–**C**) and molars with abnormal pattern in *Evc2* P0 mutants (**D**–**F**) were reconstructed from micro-CT scans. (Out of 10 *Evc2 P0 mutant*, 2 with abnormal molar patterning. The mutant with represented abnormal molar patterning is shown.).

**FIGURE 3:**
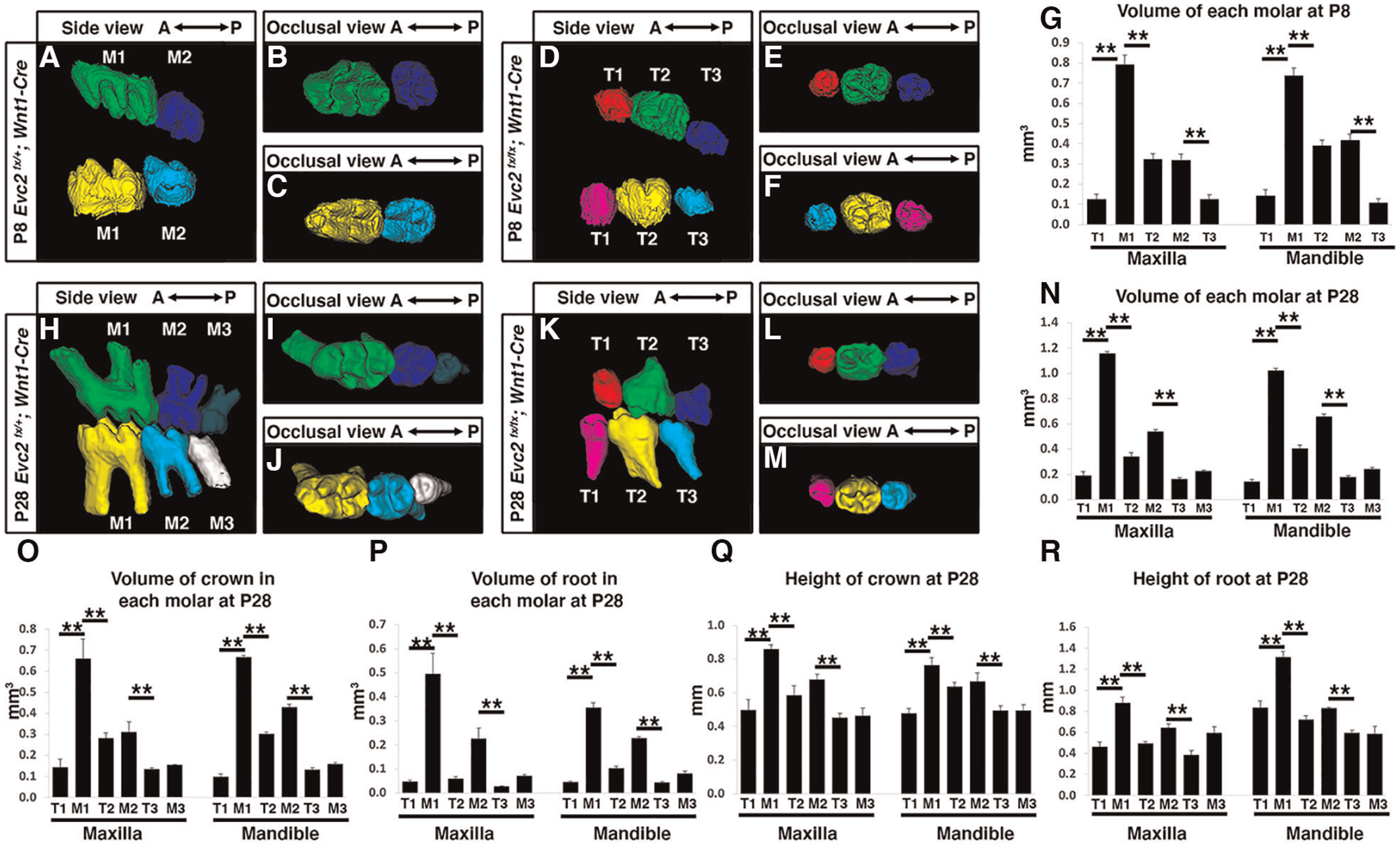
Mandible molars at P8 from controls (**A**–**C**) and *Evc2 Wnt1*- mutants (**D**–**F**) were reconstructed from micro-CT scans. The volume of each molar at P8 were quantified and shown in (**G**). Mandible molars at P28 from controls (**H**–**J**) and *Evc2 Wnt1* mutants (**K**–**M**) were reconstructed from micro-CT scans. The volume of each molar at P28 (**N**), the volume of crown (**O**), the volume of root (**P**), the height of crown (**Q**), and the height of root (**R**) were quantified and shown. For molar volumes at P8, *n* = 4, for maxilla, M1 vs T1, *p* < 0.01; M1 vs T2, *p* < 0.01; M2 vs T3, *p* < 0.01; for mandible, M1 vs T1, *p* < 0.01; M1 vs T2, *p* < 0.01; M2 vs T3, *p* < 0.01. For tooth volume, crown volume, root volume, height of crown, height of root, at P28, for maxilla, M1 vs T1, *p* < 0.01; M1 vs T2, *p* < 0.01; M2 vs T3, *p* < 0.01; for mandible, M1 vs T1, *p* < 0.01; M1 vs T2, *p* < 0.01; M2 vs T3, *p* < 0.01.

Although tooth development involves coordinated epithelial-mesenchymal interactions, we previously reported that dental epithelia-specific *Evc2* deletion did not lead to overt dental abnormalities (26). We therefore investigated the role of *Evc2* on molar patterning using two *Cre* lines, *P0-Cre* and *Wnt1-*Cre, that specifically deleted *Evc2* within neural crest cells that give rise dental mesenchymal tissues. At P28, we observed no apparent body weight differences, suggesting that there is no apparent delay in development due to *Evc2* loss of function within neural crest derived tissues. *P0-Cre* mediated deletion of *Evc2* (referred to as *Evc2 P0* mutant hereafter) resulted in mice that recapitulated the abnormal molar pattern of *Evc2* global mutants only 20% of the time (2 out of 10 mutant mice examined) (Figures 2A–C vs. 2D–F). This is in direct contrast to the 100% penetrance (12 out of 12 mutant mice examined) of abnormal molar patterning observed in both P8 (Figures 3A–C vs. 3D–F) and P28 (Figures 3H–J vs. 3K–M) mice with *Wnt1-Cre* mediated deletion of *Evc2* (referred to as *Evc2 Wnt1* mutant hereafter).

Volumetric quantification of maxillary and mandibular molars in *Evc2 Wnt1* mutants corroborated molar pattern abnormalities and indicated that M1 was larger than either T1 or T2 and that M2 was larger than T3 at both the P8 and P28 timepoints (Figures 3G,N, respectively). Similar trends were found for crown volume, root volume, crown height, and root height in *Evc2 Wnt1* mutants at P28 (Figures 3O–R). Most importantly, phenotypic similarities between mice with mesenchyme-specific loss of *Evc2* function and *Evc2* global mutants suggests that *Evc2* in mesenchymal tissues plays a critical role in determining molar patterning and morphology.

### T1 and T2 on *Evc2* mutant mandibles are at the same development stage

**FIGURE 4:**
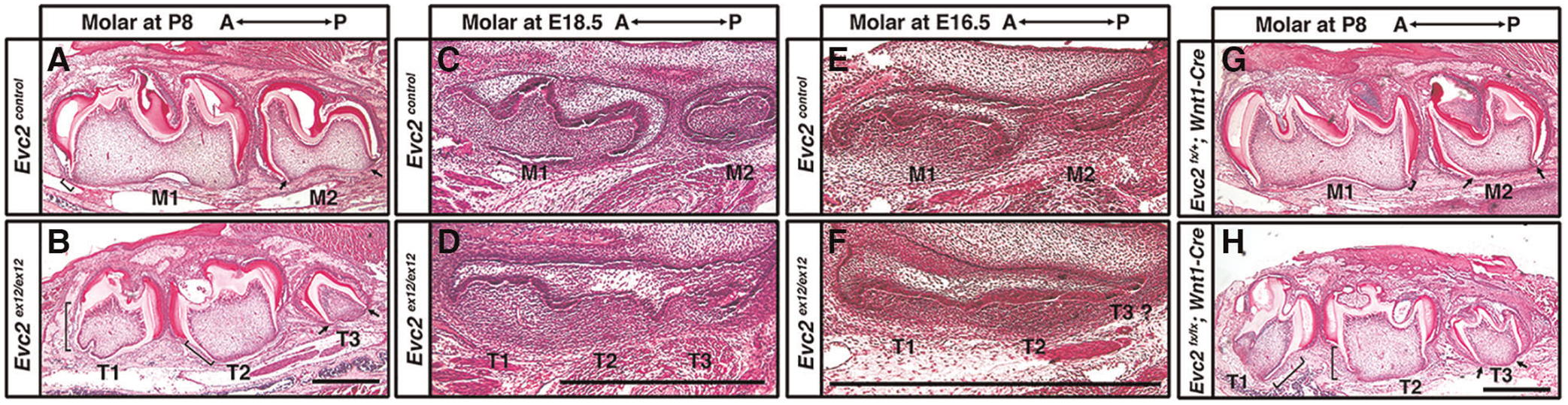
Histologic assessment of molars. Molars from control and *Evc2* mutant at P8 (**A**,**B**), E18.5 (**C**,**D**), E16.5 (**E**,**F**), molars from controls and *Evc2 Wnt* mutant at P8 (**H**,**I**) were sectioned, stained for Hemoxylin and Eosin. Bar = 100 μm. (*n* = 4). Brackets indicate the root and arrows indicate no root formation.

Crown formation precedes root formation and elongation during tooth development. Because the process of tooth maturation in mice predictably occurs in an anterior to posterior fashion, we therefore investigated the identity of accessory molar buds in *Evc2* global mutants *via* histological means. Roots were fully formed in M1 but rudimentary in M2 of control mice at P8 (Figure 4A brackets and arrows, respectively). By contrast, roots were fully formed in both T1 and T2 but rudimentary in T3 of *Evc2* global mutants at P8 (Figure 4B brackets and arrows, respectively). This suggests that molars M1, T1, and T2 were at a similar and more advanced stage of development than molars M2 and T3.

Postnatal observations regarding tooth developmental progression were corroborated *via* further analyses at embryonic day E18.5. Not only was M1 predictably more developed (bell stage) than M2 (late cup/early bell) in control mice (Figure 4C), but T1 and T2 in *Evc2* global mutants were at the same stage, albeit slightly delayed compared to M1 (Figure 4D). The T3 molar in *Evc2* global mutants was also at a comparable, though slightly delayed, stage of development to M2 in control mice (Figure 4D). When analyzed at the earlier E16.5 timepoint, M1, which was at late cup stage/early bell stage was still more developed than M2, which was at the cup stage in control mice (Figure 4E). Both T1 and T2 (cup stage) in *Evc2* global mutants were a bit delayed than M1, and T3 was a bit delayed than M2 (Figure 4F). Because anterior molars are known inducers of posterior molar formation (36), the presence of similarly staged structures in both *Evc2* global and *Evc2 Wnt1* mutants (Figures 4G,H) suggests the presence of a supernumerary anterior tooth. We did not see evidence of T4 formation (corresponding to M3 in controls) in *Evc2* global mutants or *Evc2 Wnt1* mutants. These observations are consistent with micro-CT analysis of *Evc2 Wnt1-Cre* mutants that *Evc2* loss of function within dental mesenchyme phenocopies the molar patter abnormalities in *Evc2* global mutants.

### Elevated WNT signaling and compromised Hedgehog signaling were observed in molars in *Evc2* mutant mice

**FIGURE 5:**
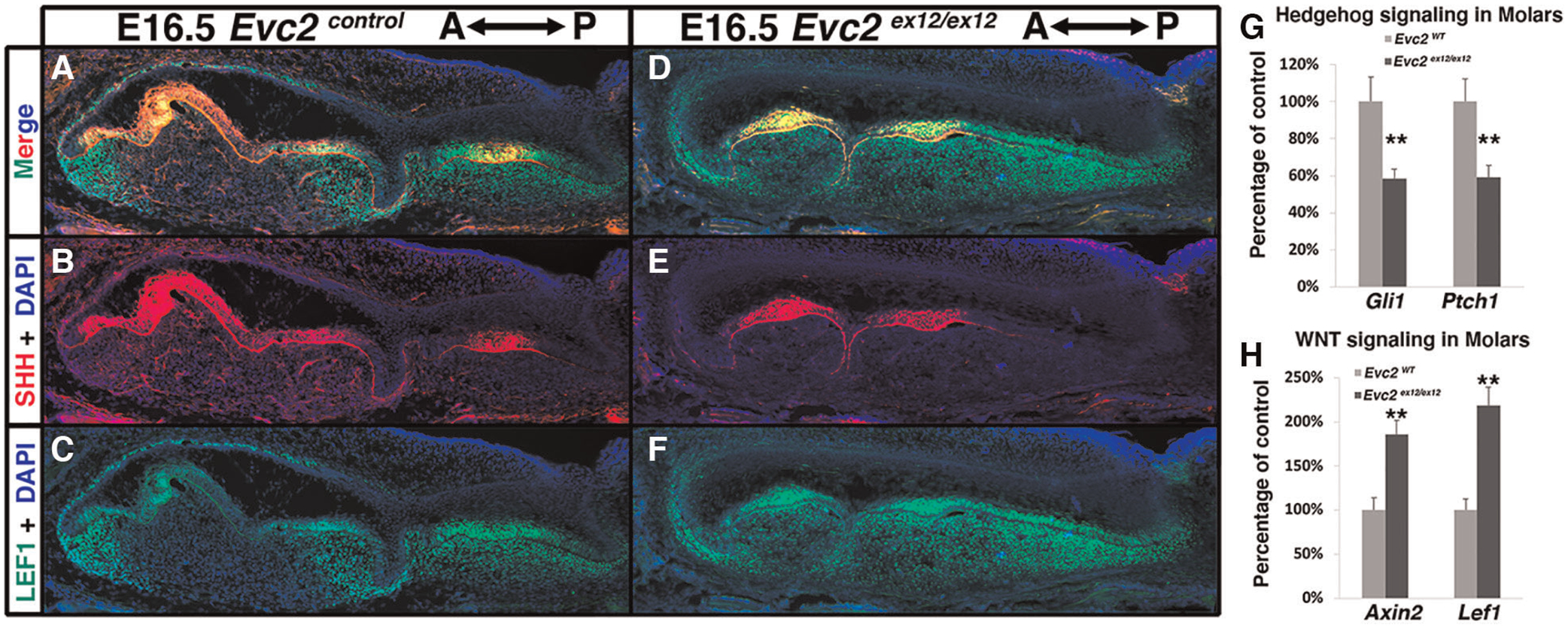
*Evc2* mutation leads to decreased Hedgehog signaling and increased WNT signaling at E16.5 and later. Mandible molars of controls (**A**–**C**) and *Evc2* mutants (**D**–**F**) were immunolabeled with LEF (**C**,**F**) for reading out WNT signaling and SHH (**B**,**E**). Hedgehog signaling (**G**) and WNT signaling (**H**) in molars were quantified through assessing the *Gli1*, *Ptch1*, *Axin2* and *Lef1* levels through q-RT-PCR. Percentage of controls were calculated and are shown in G and H. (*n* = 4; **, *p* < 0.01).

Previous studies have indicated that elevated WNT signaling and decreased Hedgehog signaling leads to abnormal molar patterning and supernumerary tooth formation in mice (37, 38). We therefore investigated WNT signaling changes in E16.5 *Evc2* global mutant molars using LEF1 as a readout of active WNT signaling. Compared to controls, we observed more cells labeled with LEF1 within the dental mesenchyme of *Evc2* global mutant molars (Figures 5A,C,D,F). Additionally, the distribution pattern of SHH corroborates our earlier histologic assessment wherein M1 is in the bell stage, while M2, T1, and T2 are in the cup stage, respectively at E16.5 (Figures 5B,E). Changes in overall Hedgehog signaling and WNT signaling levels were assessed from dissected M1 and T1 tooth germs at E18.5. qRT-PCR analyses indicated that Hedgehog signaling levels measured by expression of *Gli1* and *Ptch1* were lower in T1 than M1 (Figure 5G), despite comparable levels of SHH between two genotypes (Figures 5B,E) and that WNT signaling measured by levels of *Axin2* and *Lef1* was elevated in T1 than M1 (Figure 5H).

**FIGURE 6:**
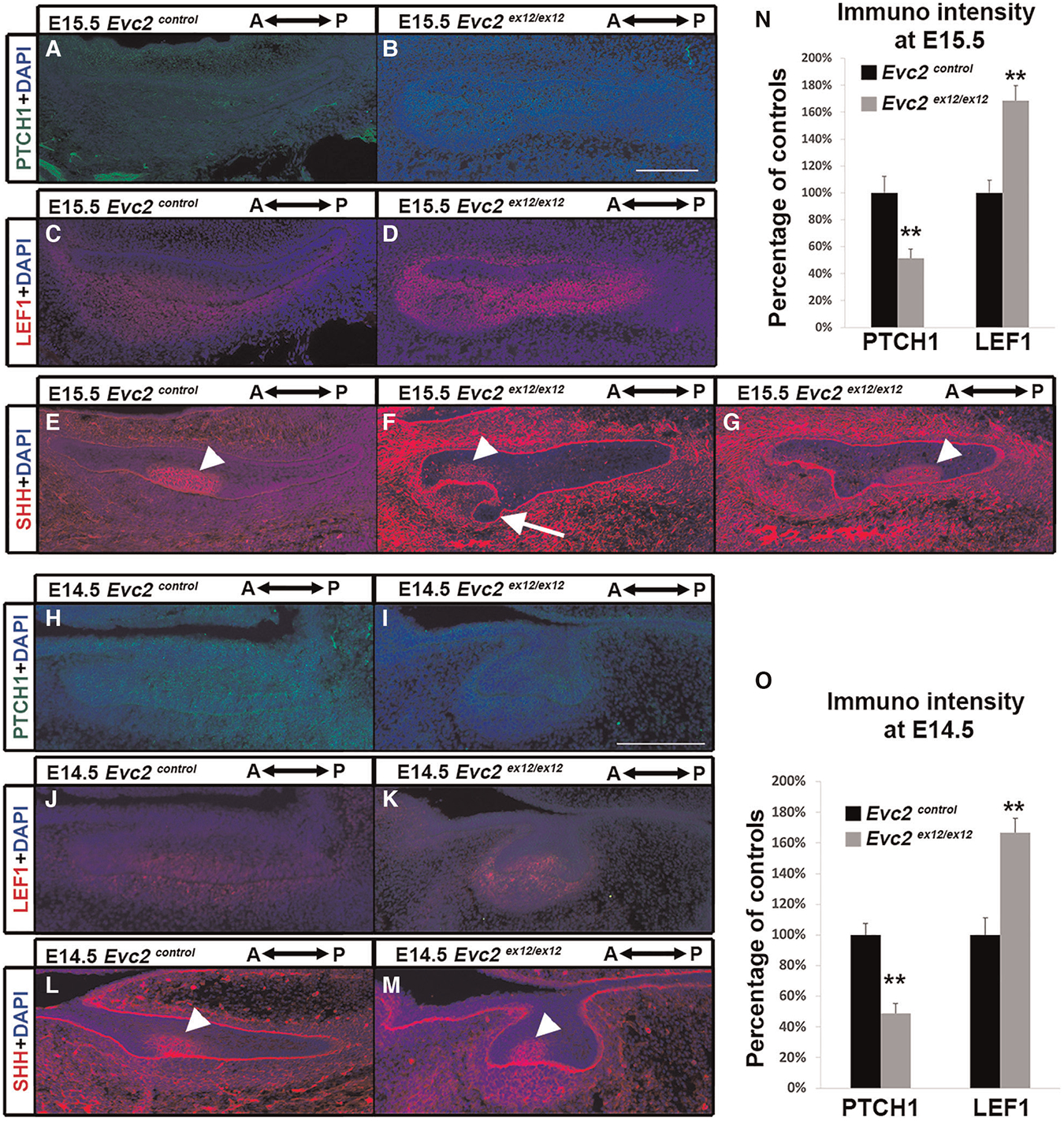
*Evc2* mutation leads to decreased Hedgehog signaling and increased WNT signaling at E14.5 and E15.5. Mandible molars from E15.5 (**A**–**G**) and E14.5 (**H**–**M**) were immunolabeled with PTCH1 (A,B,H,I) for reading out Hedgehog signaling; LEF1 (**C**,**D**,**J**,**K**) for reading out WNT signaling; and SHH (**E,F,G, L,M**) for indicating enamel knot. In *Evc2* mutants, two enamel knots were not on the same section plain. Thus, two sections with enamel knot were shown in F and G separately. The immunosignal for PTCH1 and LEF1 from E15.5 molars were quantified and shown in N. The immunosignal for PTCH1 and LEF1 from E15.5 molars were quantified and shown in O. (*n* = 4; **, *p* < 0.01). White arrow indicates abnormal shaped epithelia. White arrowheads indicate enamel knots.

To understand at which development stage the compromised Hedgehog signaling and elevated WNT signaling leads to supernumerary anterior tooth formation, we then examined the Hedgehog and WNT signaling at E15.5. Comparing to controls, we observed lower intensity of PTCH1 immuno-signals and higher intensity of LEF1 immuno-signals in both dental epithelia and dental mesenchyme (Figures 6A–D,N), suggesting that compromised Hedgehog signaling and elevated WNT signaling are already present at this stage. In contrast the controls that show only 1 enamel knot at this stage (Figure 6E), we detected two enamel knots in *Evc2* mutants (4 out of 4 mutants). In addition to the one found in the middle of tooth germ (Figure 6G), the second one was identified at an anterior location (Figure 6F, F and G are from the same tooth germ but different plain). Associated with the additional enamel knot, we observed abnormal shape of dental epithelia in *Evc2* mutant (Figure 6F), suggesting that the supernumerary tooth starts to form anterior to the M1 at E15.5 in *Evc2* mutants.

Similar to E15.5, we detected both compromised Hedgehog signaling and elevated WNT signaling in *Evc2* mutants at E14.5 (Figures 6H–K,O). However, we detected only 1 enamel knot in both controls and *Evc2* mutants (Figures 6L,M). These observations suggest that compromised Hedgehog signaling and elevated WNT signaling induce abnormal molar patterning between E14.5 and E15.5. The observation of abnormal shaped epithelia tissue at E15.5, not at E14.5 support the idea that initial abnormal patterning occurs between E14.5 and E15.5.

**FIGURE 7:**
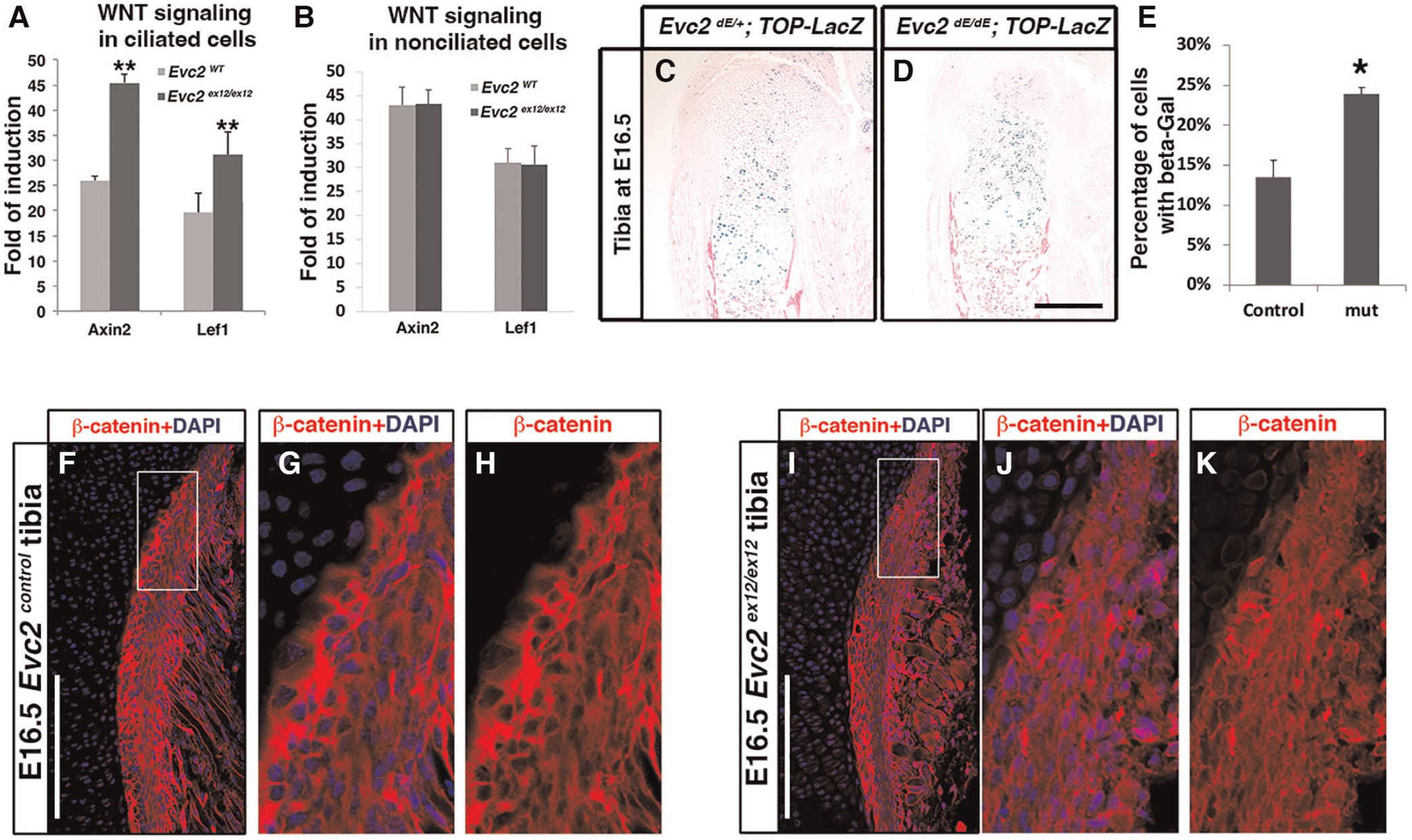
Ciliated (**A**) and non-ciliated (**B)** MEFs from controls or *Evc2* mutants were treated with WNT3A. The induction of WNT signaling was calculated and shown. WNT signaling within tibia were evaluated with a TOP-gal reporter in controls (**C**) and *Evc2* mutants (**D**). The number of cells positive for beta-gal were quantified and shown in (**E)** (*n* = 3, *p* < 0.01). E16.5 tibia from controls (**F**–**H**) and *Evc2* mutants (**I**–**K**) were immunolabeled with beta-catenin. White boxes in F and I were shown as enlarged perichondrium for controls (**G**,**H**) or *Evc2* mutants (**J**,**K**). Bar = 100 μm.

Previous studies have demonstrated that primary cilia play a negative role in regulating WNT signaling (39). Since EVC2 is a ciliary-located protein, we evaluated whether *Evc2* loss of function could lead to elevated cellular response to WNT ligands. Using isolated mouse embryonic fibroblasts (MEFs) cultured in a low serum condition to prompt ciliogenesis (ciliated cells), we quantified both *Lef1* and *Axin2* as readouts of the levels of WNT signaling induced by WNT3A. Under these conditions, we observed elevated WNT signaling in ciliated MEFs with an *Evc2* mutation (Figure 7A). In contrast, for cells cultured in under normal serum conditions (non-ciliated cells), we did not observe any changes in response to WNT ligand between *Evc2* mutants and controls. These studies indicate that primary cilia function to negatively regulate WNT signaling and that EVC2 protein within primary cilia is responsible, at least in part, for this function.

Finally, we then examined if there are other tissues in *Evc2* mutant mice with elevated WNT signaling. Using TOP-gal reporter mice, we detected an increased number of X-gal-stained cells within *Evc2* mutant growth plates compared to controls (Figures 7C–E). Similarly, WNT signaling was elevated within the perichondrium of the growth plates in *Evc2* mutants. In E16.5 tibia, we observed an elevated number of cells with nuclear located beta-catenin in the perichondrium of *Evc2* mutants (Figures 7F–K). This observation of elevated WNT signaling in multiple tissues of *Evc2* mutant mice supports the idea that *Evc2* loss of function potentially leads elevated response to WNT ligand in developing tooth, thereby contributing to abnormal molar patterning found in *Evc2* mutant mice.

## Discussions

Because hypodontia and supernumerary teeth are reported in individuals with EVC syndrome (28), we sought to understand the mechanisms underlying molar pattern alterations using an animal model of disease, especially *Evc2* mutant mice we have generated. Our studies indicate that *Evc2* within the dental mesenchyme plays a critical role in determining molar patterns. In addition, our *in vitro* and *in vivo* studies present evidence of compromised Hedgehog and elevated WNT signaling due to *Evc2* loss of function mutation, both of which possibly contribute to the abnormal molar patterns in *Evc2* mutant mice.

We utilized two Cre lines to understand the function of *Evc2* in leading to the abnormal molar formations. We did so because it was previously reported that *Wnt1-Cre* transgene influence mid-brain development (40). Though *Wnt1-Cre* transgene leads to increased WNT signaling, it is unlikely that ectopically increased WNT signaling by the transgene leads to the abnormal tooth formation as we demonstrated in this report, because 1) previous report indicated that the regions affected by *Wnt1-Cre* transgene is restricted within mid-brain and 2) in our analysis of mice with *Wnt1-Cre* transgene but no *Evc2* mutations do not show abnormal tooth patterning. Our previous studies indicate a largely overlapped but unique regions of neural crest derived tissues targeted by these two lines (41). Relevant to the current studies, *Wnt1-Cre* leads to almost 100% deletion efficiency in dental mesenchyme; in contrast, in addition to sparsely targeting into the dental epithelia, *P0-Cre* leads to about 90–95% of deletion efficiency in dental mesenchyme (21). Since *Evc2* loss of function only partially compromise Hedgehog signaling, the 90–95% deletion efficiency by *P0-Cre* may lead to decreased Hedgehog signaling, but just not enough to lead to abnormal tooth patterning.

Although the total number of molars in *Evc2* mutants is the same as in controls, we present multiple lines of evidence suggesting that the identity of each molar in *Evc2* mutants is different from controls. For example, both histological assessment and micro-CT analysis of *Evc2* mutant mandible molars at P8, E18.5, and E16.5 indicate that T1 and T2 are in the same development stage. In contrast, it is known that during molar development, the stage of M2 is around 2 days behind that of M1. The similarity of the development stage of T1 and T2 in *Evc2* mutant mandible molars suggests that T1 is a supernumerary tooth. Previous studies suggest that a variety types of abnormal signaling in tooth development leads to abnormal molar patterning. For example, increased FGF signaling due to *Spry2* or *Spry4* loss of function leads a supernumerary tooth formation anterior of M1 (42). More detailed mechanistic studies suggest that during normal molar development, successful fusion of the enamel knot from the premolar R2 with enamel knot from M1 leads to formation of one tooth germ for presumptive M1. In contrast, a failed fusion of an enamel knot from R2 with an enamel knot from M1 leads to the premolar persistence during development and a supernumerary tooth formation (17, 43–45). In this case, the existence of supernumerary tooth has been reported as early as at E14.5 (42). Additionally, *Gas1* loss of function mutant embryos form supernumerary tooth (38). Gene *Gas1* encodes a positive regulator for Hedgehog signaling. *Gas1* mutant embryos have two molars formed at the same stage during molar development. Based on this, the authors claim that the most anterior tooth is a supernumerary tooth. A similar abnormal molar patterning was reported in studies in *Sosdc1* mutant mice (37). Gene *Sosdc1* encodes a protein that functions as a negative regulation of both BMP signaling and WNT signaling through competitive bind with both BMP ligands and WNT ligands. However, previous studies indicate that the elevated WNT signaling, but not the elevated BMP signaling, leads to the formation of a supernumerary tooth anterior of M1 (37). In both above cases, the supernumerary tooth is believed as the result of continuous growth of R2 due to abnormal signaling (37, 38).

In *Evc2* mutant mandible molars, we detected decreased Hedgehog signaling, elevated WNT signaling. Particularly, the sagittal view of mandible molars is in a distinct shape in comparing to molars in *Gas1* mutants or *Sosdc1* mutants. The distinct molar shape is likely due to both decreased Hedgehog signaling and elevated WNT signaling, which showcases a unique model to understand the supernumerary tooth formation under pathologic conditions. With the presented data, it is possible that the molar at E14.5 represented a continuously developed R2 and M1 development is delayed till E15.5. Alternatively, it is possible that compromised Hedgehog signaling and increased WNT signaling leads to formation of additional enamel knot and abnormal dental epithelia invagination. Characterization of the mechanism leading to supernumerary tooth in *Evc2* mutant will rely on future detailed analysis to delineate the identity of molar at E14.5 in *Evc2* mutants.

In this study, we provide evidence that elevated WNT signaling potentially contribute to supernumerary tooth formation. Multiple previous studies suggest that elevated WNT signaling within dental epithelia lead to many misshaped ectopic tooth formations (46–48). However, in contrast to previous report, we demonstrated that dental mesenchyme-specific *Evc2* disruption results in the abnormal molar patterning and supernumerary tooth formation, because *Evc2* loss of function mediated by *Wnt1-Cre* phenocopies the abnormal tooth patterning in *Evc2* global mutants. The impact of moderately elevated WNT signaling within dental mesenchyme on supernumerary tooth formation is an interesting future direction.

The molar patterning in *Evc2/Limbin* mutant mice phenocopies the pattern of mandible molars in *Evc* mutant mice (29). This observation is consistent with the findings from molecular studies showing that interaction between EVC and EVC2 are mutually required for localization at the bottom of primary cilia (19, 20). At the bottom of primary cilia, a protein complex formed by EVC, EVC2, and SMO are required for a full activation of Hedgehog signaling. Thus, either EVC loss of function, EVC2 loss of function, or both EVC and EVC2 loss of function lead to absence of EVC-EVC2 complex at the bottom of primary cilia, which leads to compromised induction of Hedgehog signaling. Particularly during molar development in *Evc2* mutant embryos, *Evc2* loss of function leads to compromised response to Hedgehog ligand and the subsequent decreased Hedgehog signaling, which is a potential reason leading to the abnormally molar patterning in *Evc2* mutant embryos. Overall, our finding in the patterning of *Evc2* mandible molars are consistent with clinical signs in EVC syndrome and the molecular biological finding of EVC-EVC2 protein complex.

Our *in vitro* and *in vivo* studies suggest that loss of *Evc2* leads to cell autonomously increased response to WNT ligand. This is primary cilium dependent. In fact, there are multiple studies suggesting that primary cilium negatively regulates the induction of WNT signaling, although the detailed mechanism remains elusive (39, 49). Our studies indicate that EVC-EVC2 protein complex is a part of the protein machinery within primary cilium that fine-tunes the WNT signaling. Future investigation on the function of EVC-EVC2 in inducing WNT signaling will provide an in depth understanding on the primary cilium involved WNT signaling regulation.

Overall, our *in vitro* and *in vivo* studies provide evidence of both decreased Hedgehog signaling and WNT signaling in the molars of *Evc2* mutant embryos. Both types of abnormal signaling potentially contribute to the abnormal molar patterning in *Evc2* mutant embryos. Taken together, our studies implicate the potential pathological mechanisms leading to the supernumerary tooth formation in the rare disease, EVC syndrome.

## Data Availability

The original contributions presented in the study are included in the article/Supplementary Material, further inquiries can be directed to the corresponding author/s.
